# Mesenchymal Stromal Cell-Derived Extracellular Vesicles for Reversing Hepatic Fibrosis in 3D Liver Spheroids

**DOI:** 10.3390/biomedicines12081849

**Published:** 2024-08-14

**Authors:** Giulia Chiabotto, Armina Semnani, Elena Ceccotti, Marco Guenza, Giovanni Camussi, Stefania Bruno

**Affiliations:** Department of Medical Sciences, University of Torino, 10126 Torino, Italy; giulia.chiabotto@unito.it (G.C.); armina.semnani@unito.it (A.S.); elena.ceccotti@unito.it (E.C.); giovanni.camussi@unito.it (G.C.)

**Keywords:** mesenchymal stromal cells, extracellular vesicles, liver fibrosis, liver spheroids, hepatic stellate cells, collagen I, chemically defined media

## Abstract

Hepatic fibrosis, arising from prolonged liver injury, entails the activation of hepatic stellate cells (HSCs) into myofibroblast-like cells expressing alpha-smooth muscle actin (α-SMA), thereby driving extracellular matrix deposition and fibrosis progression. Strategies targeting activated HSC reversal and hepatocyte regeneration show promise for fibrosis management. Previous studies suggest that extracellular vesicles (EVs) from mesenchymal stromal cells (MSCs) can suppress HSC activation, but ensuring EV purity is essential for clinical use. This study investigated the effects of MSC-derived EVs cultured in chemically defined conditions on liver spheroids and activated HSCs. Umbilical cord- and bone marrow-derived MSCs were expanded in chemically defined media, and EVs were isolated using filtration and differential ultracentrifugation. The impact of MSC-EVs was evaluated on liver spheroids generated in Sphericalplate 5D™ and on human HSCs, both activated by transforming growth factor beta 1 (TGF-β1). MSC-EVs effectively reduced the expression of profibrotic markers in liver spheroids and activated HSCs induced by TGF-β1 stimulation. These results highlight the potential of MSC-EVs collected under chemically defined conditions to mitigate the activated phenotype of HSCs and liver spheroids, suggesting MSC-EVs as a promising treatment for hepatic fibrosis.

## 1. Introduction

Liver fibrosis is a chronic condition characterized by the progressive scarring of liver tissue caused by ongoing liver damage. The excessive accumulation of extracellular matrix (ECM) in the liver can result from various factors, such as viral infections, alcohol consumption, metabolic dysfunction-associated liver disease, cholestatic liver disease, and autoimmune hepatitis.

The pathogenesis of liver fibrosis is complex and involves the release of proinflammatory cytokines, the recruitment of hepatic macrophages, and the activation of hepatic stellate cells (HSCs) [1]. HSCs are essential in the development of liver fibrosis because they undergo a phenotypic shift from inactive vitamin A-storing cells to myofibroblasts able to proliferate and produce ECM components [2]. Activated HSCs are characterized by the expression of alpha-smooth muscle actin (α-SMA) and the synthesis of ECM proteins, including collagen alpha 1 type I (COL1A1), which build up in the liver and drive the progression of fibrosis [3,4].

Among the various chronic liver diseases, liver fibrosis poses a significant global health issue due to its high prevalence and substantial impact on disease prognosis and quality of life [5,6]. Indeed, the progression of liver fibrosis can lead to cirrhosis, which causes approximately 1 million deaths annually worldwide [7]. Furthermore, approximately 80–90% of patients with hepatocellular carcinoma have a history of liver fibrosis [8,9]. Early diagnosis and treatment of liver fibrosis are crucial steps to mitigate the global health burden. Although numerous antifibrotic drugs have undergone in vivo testing in recent years, none have been approved for clinical use [10]. However, promising new approaches, such as stem cell-based regenerative medicine and nanotherapy, have emerged as potential treatments for fibrosis [11].

Mesenchymal stromal cells (MSCs) are fibroblast-like cells that can differentiate in the osteogenic, chondrogenic, and adipogenic senses [12]. Furthermore, MSCs exhibit immunosuppressive, anti-inflammatory, and antifibrotic properties [13]. In recent decades, MSCs have been discovered in various organs, including the bone marrow (BM-MSCs) and umbilical cord (UC-MSCs), and their beneficial effects have been demonstrated in lung damage, myocardial infarction, diabetes, and liver cirrhosis [14]. Despite the numerous advantages of MSCs, their administration may present some drawbacks. For instance, genetic instability in vitro and the risk of tumorigenesis may limit their clinical application in patients [15].

MSCs mainly exert their therapeutic effects via a paracrine mechanism, releasing various cytokines, growth factors, and extracellular vesicles (EVs) [13]. The term EV encompasses a diverse group of membrane-bound nanoparticles, including apoptotic bodies (1–5 μm in size), microvesicles or ectosomes (100–1000 nm), and exosomes (50–150 nm). Through transferring proteins, lipids, and various RNA species to recipient cells, EVs can induce epigenetic changes in damaged tissue cells, thereby fostering their regeneration [16].

In the last decades, EVs derived from MSCs (MSC-EVs) have shown enormous therapeutic potential for various chronic diseases, in particular, to counteract hepatic fibrosis, mainly by reducing inflammation and collagen deposition [17,18]. Recently, we have shown that EVs isolated from human liver stem cells (HLSCs), which are liver-resident MSC-like cells, can reduce the expression of specific fibrosis markers and influence the lncRNA landscape in the liver of mice with nonalcoholic steatohepatitis (NASH) [19,20]. Additionally, in an in vitro model of liver fibrosis based on transforming growth factor beta 1 (TGF-β1)-activated HSCs cultured in a monolayer, we demonstrated that EVs derived from HLSCs and BM-MSCs can effectively downregulate the expression of αSMA and COL1A1 in activated HSCs [21]. The observed antifibrotic activity could be ascribed to the different components shuttled by the MSC-EVs, in particular, to the presence of specific miRNAs, such as miR-146a-5p [21], miR-378c [22], and miR-199a-5p [23,24].

A significant drawback of traditional 2-dimensional (2D) cell culture is the lack of the surrounding microenvironment and interactions between different types of liver cells, which makes it difficult to accurately reproduce the pathophysiological mechanisms of liver fibrosis [25]. However, the recent development of 3-dimensional (3D) culture systems has made it possible to study the intricate interactions between the various cells involved in the progression of fibrosis, as well as the architectural and functional changes that are characteristic of liver fibrosis, such as the accumulation of ECM proteins induced by HSCs [26]. Liver organoids and spheroids of different cellular compositions have been successfully used to reproduce a variety of hepatobiliary diseases in vitro to study hepatotoxicity and screen antifibrotic drugs [27].

This study aims to develop different 3D models of liver fibrosis consisting of hepatocytes and HSCs. Using these 3D models, we assessed the antifibrotic potential of EVs isolated from BM-MSCs and UC-MSCs cultured under chemically defined conditions.

## 2. Materials and Methods

### 2.1. Cell Culture

Human BM-MSCs and UC-MSCs were provided by RoosterBio (Frederick, MD, USA). Three different xeno-free batches of each cell source were used. After thawing, cells were seeded at a concentration of 3000 cells/cm^2^ into T75 cell culture flasks (Sarstedt, Milan, Italy), coated with 0.1 mL/cm^2^ of 2.5 µg/mL vitronectin (Gibco, Thermo Fisher Scientific, Carlsbad, CA, USA) in phosphate buffer saline (PBS), expanded in the RoosterNourish™-MSC-XF medium, and used until passage 6.

Primary hepatocytes (UpHep) purchased by Upcyte^®^ (Hamburg, Germany) were seeded at a concentration of 10,000 cells/cm^2^ into T75 cell culture flasks coated with 0.1 mL/cm^2^ of 50 µg/mL collagen type I (Gibco) in 20 mM acetic acid. The UpHep were maintained in a high-performance medium and used up to passage 2. HepG2 cells were maintained in Dulbecco’s modified Eagle’s medium (DMEM, Euroclone, Pero, MI, Italy), supplemented with 10% fetal bovine serum (FBS) and 2 nM L-Glutamine (both from Euroclone, Pero, MI, Italy). The human HSC line LX-2 [28] (Sigma Aldrich, St. Louis, MO, USA) was cultured in DMEM high glucose (4.5 g/L, Euroclone) containing 2% fetal calf serum and 2 nM L-Glutamine and used up to passage 6.

### 2.2. EV Isolation

For EV collection, BM-MSCs and UC-MSCs were seeded at densities of 3000 cells/cm^2^ and 2000 cells/cm^2^, respectively, in vitronectin-coated T75 cell culture flasks. When 70–80% confluence was reached, cells were washed with a sterile saline solution and cultured in a chemically defined RoosterCollect™-EV medium for 24 h. Subsequently, the cell supernatant was collected, centrifuged at 3000× *g* for 15 min at 4 °C, and microfiltrated with 0.22 µm filters. After removing cell debris and apoptotic bodies, the filtered medium was transferred to polycarbonate centrifuge bottles (Beckman Instruments, Brea, CA, USA) and subsequently ultracentrifuged at 100,000× *g* for 2 h at 4 °C (Beckman Coulter Optima L-100K). The resulting EVs were diluted in PBS and subjected to another round of ultracentrifugation at 100,000× *g* for 2 h at 4 °C to reduce protein contaminants. The final EV pellet was resuspended in PBS containing 1% dimethyl sulfoxide (DMSO, Sigma Aldrich) and stored at −80 °C until use in subsequent experiments.

The NanoSight NS300 device (NanoSight Ltd., Amesbury, UK) was used to evaluate the concentration and size distribution of EVs under steady flow conditions (flow rate = 30). For each EV sample, three 60 s videos were recorded, and the data were processed using Nanoparticle Tracking Analysis (NTA) 3.2 software.

EV phenotype characterization was performed by cytofluorimetric analysis, following previously established protocols [19,29,30]. Using the human MACSPlex Exosome Kit (Miltenyi Biotec, Bergisch Gladbach, Germany), approximately 1 × 10^9^ EVs from three batches, each of the BM-MSC-EVs and UC-MSC-EVs, were examined. After incubation with the MACSPlex Exosome Capture Beads and counterstaining with APC-conjugated antitetraspanins antibodies, EV samples were analyzed using a Cytoflex (Beckman Coulter). The median fluorescence intensity (MFI) of each capture bead subset was determined using CytExpert Software (version 2.0, Beckman Coulter). This was achieved by subtracting the MFI of a blank control (background fluorescence intensity) from the MFI of all 39 capture bead subsets. The resulting MFI was then normalized based on the average MFI of the exosomal markers CD63, CD81, and CD9.

The EVs’ size and integrity were evaluated using transmission electron microscopy. Approximately 3 × 10^9^ EVs were deposited onto 200 mesh nickel formvar carbon-coated grids (Electron Microscopy Science, Hatfield, PA, USA) and were allowed to adhere for 20 min, following previously established protocols [31]. Subsequently, the grids underwent PBS washing before being incubated with a 2.5% glutaraldehyde solution containing 2% sucrose, followed by thorough rinsing with distilled water. Finally, the EVs were subjected to negative staining using NanoVan (Nanoprobes, Yaphank, NY, USA) and examined with a Jeol JEM 1400 Flash electron microscope (Jeol, Tokyo, Japan).

### 2.3. Hepatic Stellate Cell Activation

LX-2 cells were synchronized in a serum-deprived medium containing 0.2% of bovine serum albumin (Sigma Aldrich) for 6 h, followed by seeding at a density of 13,000 cells/cm^2^. To induce activation, LX-2 cells were treated with TGF-β1 (Sigma-Aldrich, 10 ng/mL) for 6 h. Subsequently, activated LX-2 cells were exposed to 50,000 EVs per cell for 24 h and then lysed for RNA extraction.

### 2.4. Generation of Hepatic Spheroids

To establish hepatic spheroids, we used Sphericalplate 5D^®^ (Kugelmeiers, Erlenbach, Switzerland), a 24-well plate with specially designed wells capable of containing up to 750 spheroids each. One ml/well of medium was used to remove air bubbles from the plate before seeding. To generate the spheroids of UpHep or HepG2, 150 cells/µwell were seeded in the presence of TGF-β1 (10 ng/mL), with a total final volume of 2 mL of complete medium per well. To generate spheroids made of a co-culture of UpHep or HepG2 and LX-2 cells, cells were mixed in a ratio of 10:1 before seeding in the presence of TGF-β1. For the setup experiments, spheroids were collected on days 1, 2, 3, and 6 after seeding. In selected experiments, the spheroids were allowed to settle and form for 24 and 48 h before EV stimulation. Two doses of BM-MSC-EVs and UC-MSC-EVs were tested: 2 × 10^8^ EVs/well (EV1; 2000 EVs/cell) and 1 × 10^9^ EVs/well (EV2; 8000 EVs/cell). Spheroids were incubated with EVs up to day 6 after seeding, then washed with PBS and lysed for RNA extraction.

### 2.5. Molecular Analysis

RNA was isolated from cells using the miRNeasy mini kit (Qiagen, Valencia, CA, USA) and converted into cDNA, as described [21]. The 96-well QuantStudio 12K Flex Real-Time PCR system (Thermo Fisher Scientific) was employed for quantitative real-time polymerase chain reaction (RT-PCR), performed as previously reported [21]. Sequence-specific oligonucleotide primers (100 nM, procured from MWG-Biotech, Eurofins Scientific, Brussels, Belgium) are listed in Table 1. Relative gene expression level changes were determined using the ΔΔCt method, with TBP as the reference gene.

### 2.6. Western Blot

For protein analysis, the cells and EVs were lysed on ice, using Lysis Buffer 17 containing 10 µg/mL aprotinin, 10 µg/mL leupeptin, and 10 µg/mL pepstatin (all purchased from R&D Systems, Minneapolis, Minnesota, USA), following the manufacturer’s instructions. Protein concentration was quantified using the BCA Protein Assay Kit (Pierce™ Thermo Fisher Scientific). Proteins were separated by electrophoresis in 4–20% polyacrylamide gels and transferred onto nitrocellulose membranes, as previously described [21]. Membrane blocking was performed in Clear Milk Blocking Buffer (Pierce™ Thermo Fisher) for 2 h at room temperature, followed by overnight incubation at 4 °C with primary antibodies, all purchased from Cell Signaling Technologies (Danvers, MA, USA) and listed in Table 2. After a 1 h incubation with appropriate peroxidase-conjugated secondary antibodies (Invitrogen, Thermo Fisher Scientific), chemiluminescent signals were developed using SuperSignal™ West Femto Maximum Sensitivity Substrate (Thermo Fisher Scientific) and visualized using the Chemidoc instrument (Bio-Rad, Hercules, CA, USA).

### 2.7. Immunostaining

Six days post-seeding, spheroids were harvested from the Sphericalplate 5D^®^ and seeded in chamber slides (Corning, Glendale, AZ, USA) overnight. The day after, spheroids were rinsed with PBS and fixed with 2% paraformaldehyde for 30 min at 4 °C. After a 30 min blocking step in PBS containing 5% bovine serum albumin (Sigma Aldrich), spheroids were incubated for 1 h with a primary antibody targeting collagen I alpha 1 (Santa Cruz Biotechnology, Dallas, TX, USA). Following PBS washes, spheroids were incubated for 1 h at room temperature with an Alexa Fluor 488-conjugated secondary antibody (Molecular Probes, Invitrogen, Eugene, OR, USA). Spheroids stained only with the secondary antibody served as controls. Hoechst 33258 dye (Sigma Aldrich) was applied for nuclear staining. Fluorescent microscopy analysis was conducted using a Zeiss Apotome Microscope (Carl Zeiss International, Jena, Germany).

### 2.8. Statistical Analyses

A GraphPad Prism v.8.0 was utilized for data analysis. The results were presented as mean ± standard deviation (SD). Statistical significance was determined using ANOVA followed by Dunnett’s multiple comparisons test, with a *p*-value of <0.05 considered significant.

## 3. Results

### 3.1. Liver Spheroid Formation Using HepG2 and LX-2

**Figure 1:**
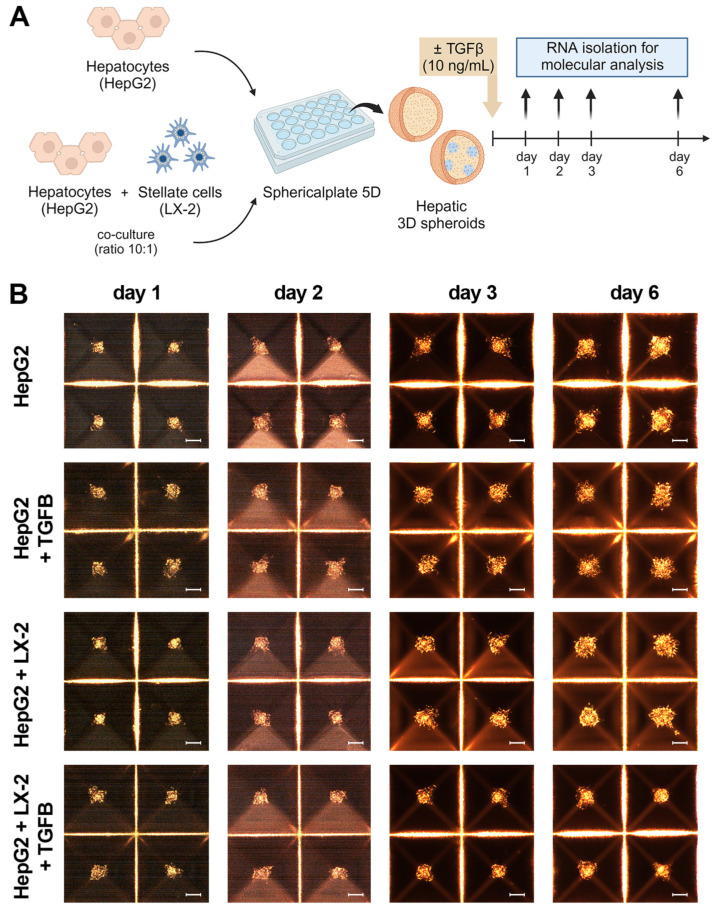

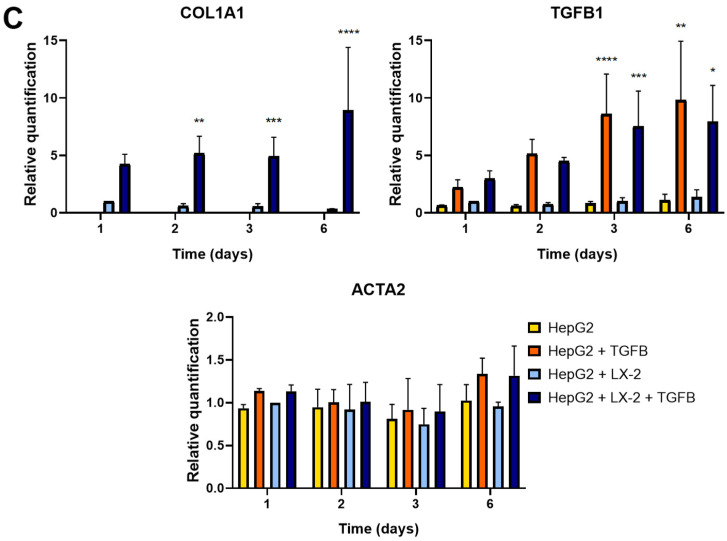
Set up of liver spheroids using HepG2 and LX-2. (**A**) Diagram illustrating the experimental setup performed in vitro to generate liver spheroids using the co-culture of HepG2 and LX-2 or HepG2 only; created with BioRender.com. (**B**) Morphological observation in light microscopy of liver spheroids from day 1 to day 6 after cell seeding (scale bar, 100 µm). (**C**) Quantitative RT-PCR analysis of profibrotic gene expression in liver spheroids up to 6 days post-seeding. Expression levels were normalized to the *TBP* reference gene. Liver spheroids of HepG2 and LX-2 not activated by TGF-β1 served as reference control. Data from at least three independent experiments were analyzed using a two-way ANOVA test: * *p* < 0.05; ** *p* < 0.01; *** *p* < 0.001; and **** *p* < 0.0001.

Liver spheroids were generated by co-culturing a hepatocyte surrogate cell line (HepG2) with human HSCs (LX-2) in a 10:1 ratio within a Sphericalplate 5D^®^ (Figure 1A). This particular culture system facilitated the rapid formation of liver spheroids. As the control, we set up liver spheroids formed only from HepG2 cells. In both models, liver cells began to aggregate within 24 h of seeding and maintained their 3D spheroid structures until the sixth day post-seeding. Over this time, aside from a slight increase in the size of spheroids, no observable changes in morphology were observed (Figure 1B).

As depicted in the workflow (Figure 1A), we induced fibrosis by exposing liver spheroids formed by the co-culture of HepG2 and LX-2 or HepG2 to TGF-β1. Morphological and molecular changes were then assessed up to the sixth day of culture, and only in the spheroids formed by the co-culture, a reduced growth was observed following TGF-β1 exposure (Figure 1B). In spheroids formed by HepG2 and LX-2, TGF-β1 treatment resulted in the increased expression of fibrotic markers *COL1A1* and *TGF-β1*, while no significant differences in *α-SMA* expression were observed (Figure 1C). Interestingly, spheroids formed exclusively by HepG2 did not express *COL1A1* per se, and TGF-β1 stimulation increased the expression levels of *TGF-β1* while not affecting *COL1A1* expression (Figure 1C). Additionally, immunofluorescence analysis using confocal microscopy confirmed the TGF-β1-induced upregulation of Collagen I expression in co-culture spheroids (Figure S1A). These findings collectively suggest that TGF-β1 can induce a fibrotic phenotype in liver spheroids generated by the co-culture of HepG2 and LX-2.

### 3.2. Liver Spheroid Formation Using UpHep and LX-2

A 3D model using primary hepatocytes was also set up. As shown in Figure S2A, in the 2D culture, UpHep appeared heterogeneous in morphology, with polygonal and spindle-shaped cells. This mixed population might include not only mature hepatocytes but also other liver cells, which have a fibroblast-like morphology and express *COL1A1* and *α-SMA* at the mRNA level, resembling the characteristics of HSCs (Figure S2B). Moreover, compared to the 2D culture, UpHep cultured as spheroids showed the reduced expression of fibrosis-associated genes (Figure S2B). This result suggests that the 3D culture allows the HSC population within UpHep to maintain a quiescent state. In contrast, the 2D culture tends to promote HSC activation, as demonstrated by the higher expression of *COL1A1* and *α-SMA*. Notably, compared to the 2D culture, UpHep cultured as spheroids showed a significant increase in the expression of the hepatocyte marker *CYP1A1* starting from the first day of the 3D culture (Figure S2C). Additionally, hepatocyte markers *albumin* and *CYP3A4* expression increased significantly in UpHep spheroids starting from the second day of culture (Figure S2C). These data indicate that a 3D culture can promote hepatocyte differentiation more effectively than a 2D culture.

**Figure 2:**
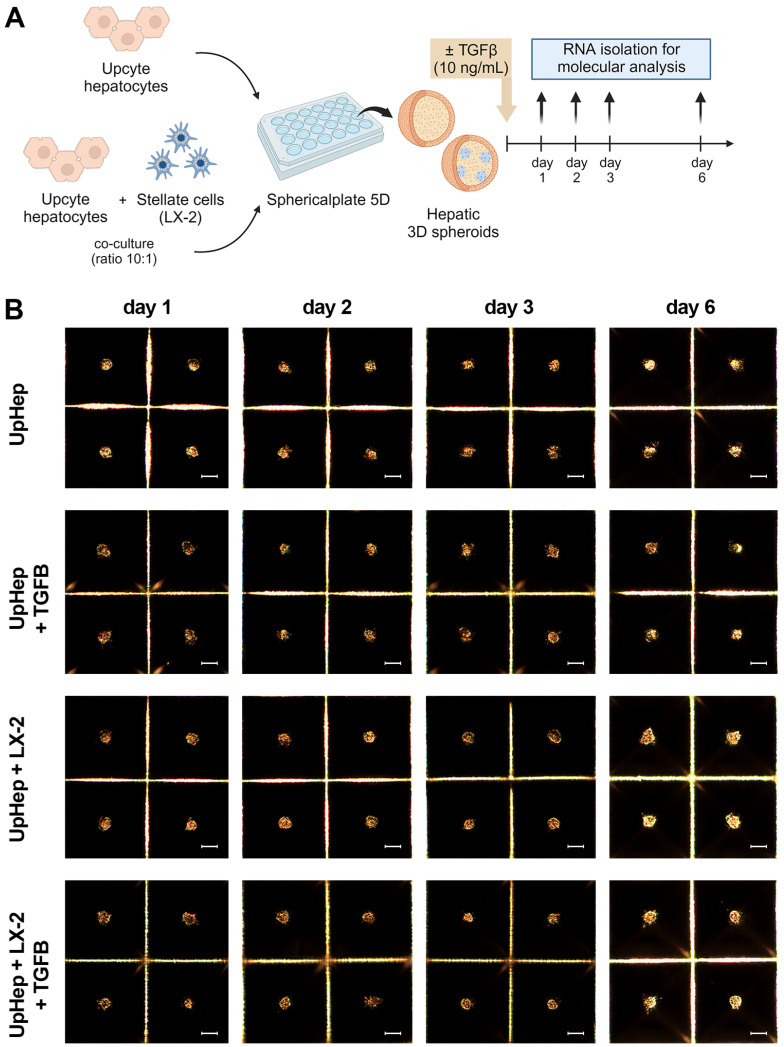

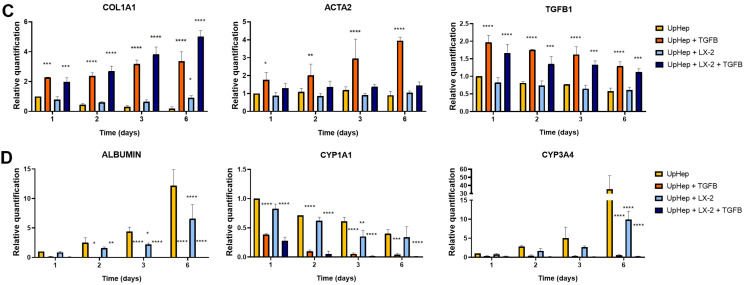
Set up of liver spheroids using UpHep and LX-2. (**A**) Diagram illustrating the experimental setup performed in vitro to generate liver spheroids using the co-culture of UpHep and LX-2 or UpHep only; created with BioRender.com. (**B**) Morphological observation in light microscopy of liver spheroids from day 1 to day 6 after cell seeding (scale bar, 100 µm). (**C**,**D**) Quantitative RT-PCR analysis evaluating the expression of profibrotic (**C**) and hepatocyte-specific (**D**) genes in liver spheroids up to 6 days after seeding. Expression levels were normalized to the *TBP* reference gene. Liver spheroids of UpHep not activated by TGF-β1 served as reference control. Data from at least three independent experiments were analyzed using a two-way ANOVA test: * *p* < 0.05; ** *p* < 0.01; *** *p* < 0.001; and **** *p* < 0.0001.

We generated liver spheroids in a Sphericalplate 5D^®^ by co-culturing UpHep with LX-2 cells at a 10:1 ratio (Figure 2A). As a control, liver spheroids consisting solely of UpHep were also prepared. Similarly, to the previous 3D model, liver cells began to aggregate within 24 h of seeding and maintained their 3D spheroid appearance until the sixth day post-seeding. No significant morphological changes were observed throughout this time in the spheroids (Figure 2B).

To induce fibrosis in the hepatic spheroids, liver cells were seeded in the presence of TGF-β1 (Figure 2A). Morphological and molecular changes were evaluated up to the sixth day of culture, and no significant changes in growth were observed following exposure to TGF-β1 (Figure 2B). Treatment with TGF-β1 resulted in the increased expression of the fibrotic markers *COL1A1* and *TGF-β1* at the mRNA level (Figure 2C). Immunofluorescence analysis of spheroids formed by UpHep confirmed an increased expression of Collagen I induced by TGF-β1 (Figure S1B). Notably, TGF-β1 stimulation increased *αSMA* expression levels exclusively in spheroids formed by UpHep, suggesting that HSCs might be present in UpHep and acquire an activated phenotype upon TGF-β1 exposure (Figure 2C).

In addition, the expression of *albumin* and other hepatocyte-specific enzymes (*CYP1A1* and *CYP3A4*) in hepatic spheroids was significantly repressed by TGF-β1 stimulation (Figure 2D). Taken together, these results indicate that TGF-β1 can induce a fibrotic phenotype in liver spheroids with an impairment in liver function, especially in those spheroids generated exclusively by UpHep, which we decided to use in subsequent experiments.

### 3.3. Characterization of EVs

**Figure 3:**
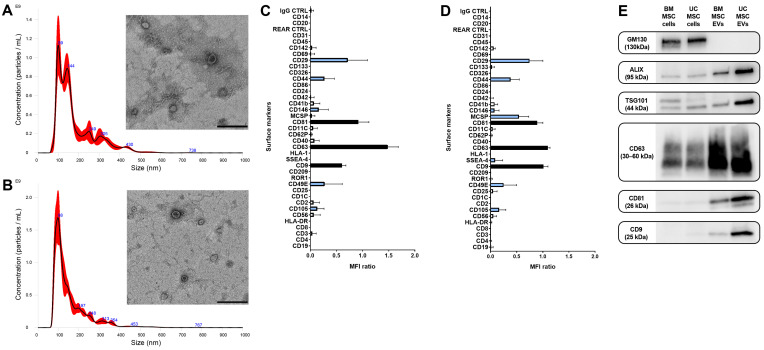
Characterization of BM-MSC-EVs and UC-MSC-EVs. (**A**,**B**) Representative graphs illustrating EV size distribution via Nanoparticle Tracking Analysis (red lines indicate the mean of the three recorded videos), alongside micrographs of transmission electron microscopy showcasing intact BM-MSC-EVs (**A**) and UC-MSC-EVs (**B**). EVs underwent negative staining using NanoVan (scale bar, 200 nm). (**C**,**D**) The molecular surface profiles of BM-MSC-EVs (**C**) and UC-MSC-EVs (**D**) were assessed via a multiplex bead-based flow cytometry assay. Exosomal markers and mesenchymal markers are indicated by black bars and light blue bars, respectively. The two graphs present a quantification of median APC fluorescence values for 39 different bead populations following background correction. Notably, no statistically significant variances were detected among three distinct preparations of BM-MSC-EVs and UC-MSC-EVs. (**E**) Representative Western blot analysis depicting the presence of exosomal markers in EVs. The cis-Golgi marker GM130, absent in EVs but expressed in cells, served as the negative control.

EVs derived from BM-MSCs and UC-MSCs were characterized in accordance with ISEV guidelines [32]. An NTA analysis revealed a mean size of EVs comparable to that of exosomes and microvesicles, while a TEM analysis confirmed that the EV preparations contained intact nanoscale particles with double membranes (Figure 3A,B). 

A flow cytometric analysis (Figure 3C,D) and Western blot analysis (Figure 3E) demonstrated that EVs expressed the typical exosomal markers ALIX, TSG101, CD9, CD63, and CD81. The mesenchymal origin of EVs was validated by the presence of surface molecules CD29, CD44, CD105, and CD146, which are characteristic of MSCs. Additionally, the expression of stem cell markers MCSP and SSEA-4 was higher in UC-MSC-EVs compared to BM-MSC-EVs. Both EV populations showed no expression of epithelial markers EpCAM/CD326 and CD24, hematopoietic markers (CD45) and endothelial markers (CD31), or human leukocyte antigens (HLA) class I and II (Figure 3C,D).

### 3.4. EVs Alleviated Fibrosis in Liver Spheroids

**Figure 4:**
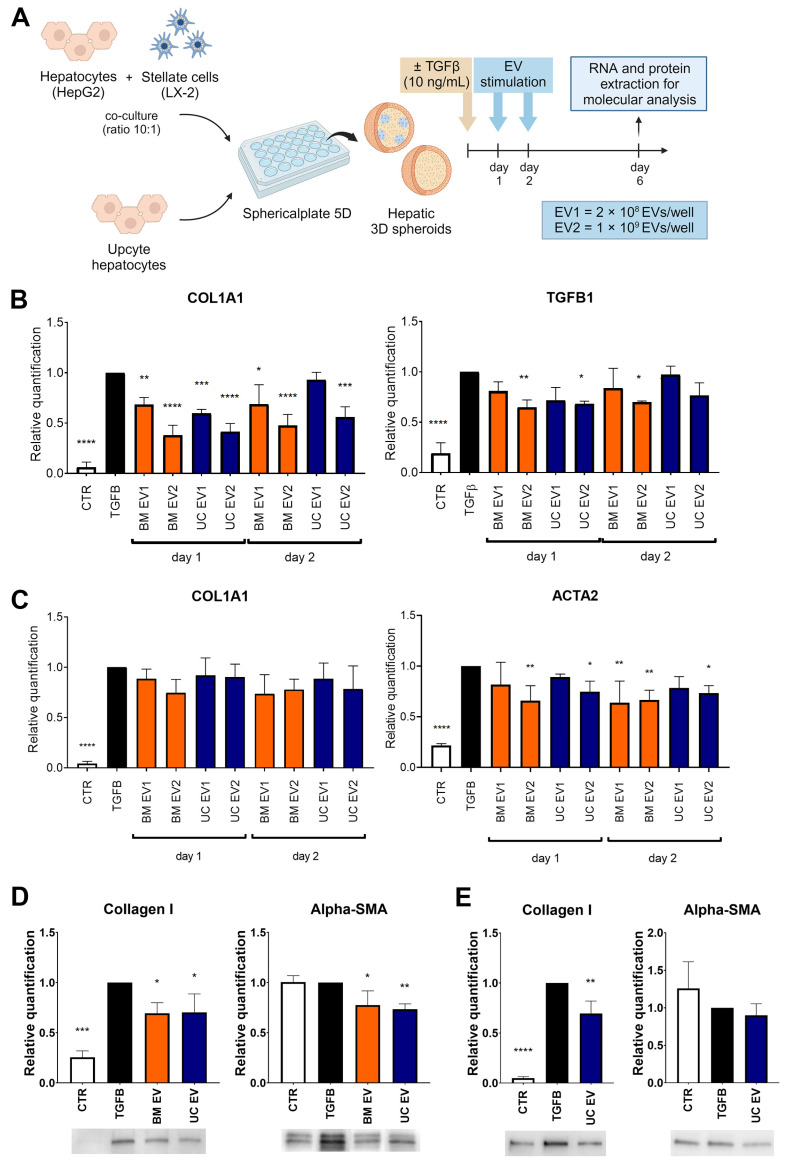
Antifibrotic effect of EVs on hepatic spheroids treated with TGF-β1. (**A**) Diagram illustrating the experimental setup conducted in vitro to assess the impact of EVs derived from BM-MSCs and UC-MSCs on spheroids generated using HepG2 and LX-2 or UpHep only; created with BioRender.com. (**B**,**C**) Quantitative RT-PCR analysis evaluating the expression of profibrotic genes in spheroids generated using HepG2 and LX-2 (**B**) or UpHep only (**C**) after stimulation with EVs obtained from BM-MSCs and UC-MSCs. Expression levels were normalized to the TBP reference gene. (**D**,**E**) Quantification of protein bands and representative images from Western blot analysis showing profibrotic markers in spheroids formed by HepG2 and LX-2 (**D**) or UpHep only (**E**). Protein band intensities were normalized to vinculin expression. All the experiments’ reference controls consisted of liver spheroids activated with TGF-β1 (10 ng/mL) but not treated with EVs. The negative control (CTR) consisted of liver spheroids cultured in the absence of TGF-β1. Data from at least three independent experiments were analyzed using a two-way ANOVA test: * *p* < 0.05; ** *p* < 0.01; *** *p* < 0.001; and **** *p* < 0.0001.

We assessed the effect of EVs on liver spheroids composed of HepG2 and LX-2 cells and on liver spheroids formed by UpHep. In both 3D models, liver spheroids were treated with two different doses of EVs (EV1 = 2 × 10^8^ EVs/well and EV2 = 1 × 10^9^ EVs/well) from BM-MSC and UC-MSC at 24 h and 48 h after seeding. Subsequently, spheroids were analyzed on the sixth day of culture (Figure 4A). The administration of EVs did not yield discernible effects on the growth status of TGF-β1-treated liver spheroids (Figure S3A). At the RNA level, treatment with the highest dose of EVs from BM-MSC and UC-MSC was able to attenuate the expression of *COL1A1* and *TGF-β1* in HepG2 and LX-2 spheroids (Figure 4B) and reduce the expression of *αSMA* in UpHep spheroids (Figure 4C). The downregulation of Collagen I and αSMA was also observed at the protein level in HepG2 and LX-2 spheroids (Figure 4D) and in UpHep spheroids (Figure 4E). These results suggest that the EVs, at the tested doses, are able to attenuate the fibrotic phenotype induced by TGF-β1 in liver spheroids. Finally, treatment with EVs did not significantly alter the expression of *albumin* and liver enzymes in TGF-β1-treated primary hepatocyte spheroids (Figure S3B).

### 3.5. EVs Mitigated the Activated Phenotype of HSCs

**Figure 5:**
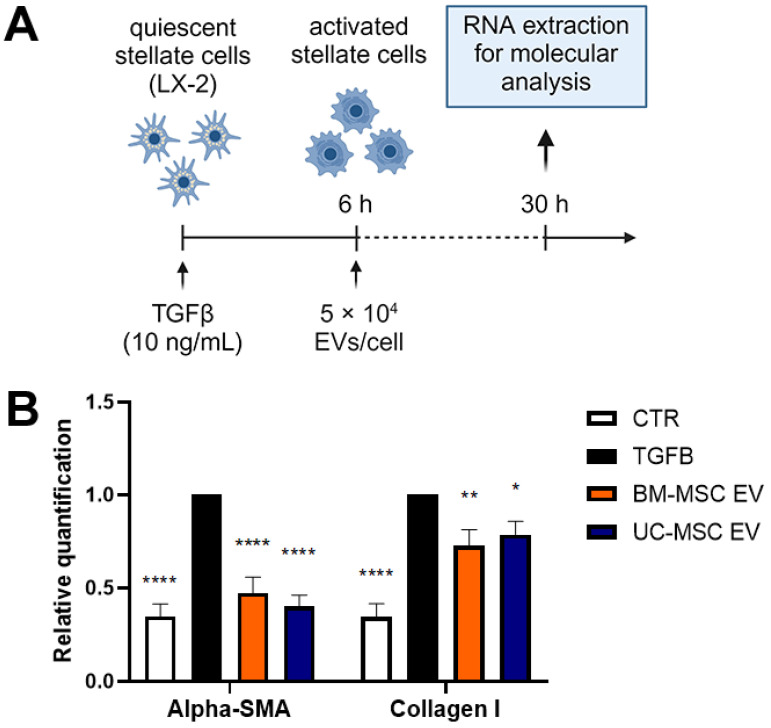
Antifibrotic effect of EVs on TGF-β1-activated LX-2. (**A**) Diagram illustrating the experimental setup conducted in vitro to assess the impact of EVs derived from BM-MSCs and UC-MSCs on LX-2 cells; created with BioRender.com. (**B**) Quantitative RT-PCR analysis of profibrotic gene expression in LX-2 cells after a 24 h incubation period with EVs obtained from BM-MSCs and UC-MSCs. Expression levels were normalized to the *TBP* reference gene. Reference control consisted of LX-2 cells activated with TGF-β1 (10 ng/mL) but not treated with EVs. The negative control (CTR) consisted of LX-2 cells cultured in the absence of TGF-β1. Data from at least three independent experiments were analyzed using a two-way ANOVA test: * *p* < 0.05; ** *p* < 0.01; and **** *p* < 0.0001.

To examine how EVs affect the activated state of HSCs, LX-2 cells activated by TGF-β1 were treated with EVs obtained from either BM-MSCs or UC-MSCs, following the procedure outlined in Figure 5A. The activation of LX-2 cells by TGF-β1 led to the increased expression of two fibrosis-associated genes, *α-SMA* and *COL1A1*. However, after incubating LX-2 cells with EVs from both BM-MSCs and UC-MSCs for 24 h, a significant decrease in the expression levels of *α-SMA* and *COL1A1* was observed (Figure 5B).

## 4. Discussion

Liver fibrosis manifests as a chronic, progressive, and irreversible condition characterized by hepatocyte injury and tissue structural remodeling. While the liver typically demonstrates regenerative capacity in response to acute injury, chronic liver insults trigger sustained inflammatory and wound-healing responses, impairing its ability to regenerate effectively [33]. Conventional anti-inflammatory treatments often fail to elicit a therapeutic response in the advanced stages of chronic liver injury. Although certain antifibrotic drugs and stem cell therapy offer limited efficacy, emerging nanomedicine-based approaches hold promise for liver fibrosis prevention and treatment [11]. Notably, EVs derived from various MSC sources have demonstrated therapeutic potential in fibrotic conditions affecting diverse organs, including the heart, kidneys, and lungs [34]. In particular, EVs from BM-MSCs [35] and UC-MSCs [36,37,38] have exhibited protective antifibrotic effects in preclinical liver fibrosis models.

Priming strategies that modulate the cellular environment of MSCs during in vitro expansion have been shown to enhance the functional attributes of MSCs and their EVs significantly. Preconditioning techniques, such as hypoxic preconditioning, exposure to proinflammatory cytokines, and 3D culture environments, can improve the therapeutic potential of MSC-EVs in addressing specific pathological contexts, such as hepatic fibrosis [39]. Hypoxic preconditioning involves culturing MSCs under low oxygen conditions, which upregulates hypoxia-inducible factor 1-alpha, leading to the secretion of EVs with enhanced regenerative capabilities [40,41]. Hypoxia-induced MSC-EVs are enriched with angiogenic factors and exhibit an enhanced capacity to promote tissue repair and angiogenesis. Exposure to proinflammatory cytokines, such as tumor necrosis factor-alpha and interferon-gamma, primes MSCs to produce EVs with improved anti-inflammatory and immunomodulatory properties [42,43]. Additionally, MSCs cultured in 3D environments, such as spheroids or scaffold-based systems, produce EVs with superior antifibrotic activity compared to those from 2D cultures, owing to the increased secretion of bioactive molecules [44]. Optimizing these priming techniques could greatly advance MSC-EV therapies for hepatic fibrosis and other fibrotic diseases.

To ensure the safety and efficacy of EVs in a future clinical context, it is necessary to eliminate all potential contamination present in the culture media. In this regard, it is preferable not to use media containing FBS or human platelet lysate, which could potentially also be a source of EVs [45]. To eliminate the unknown side effects of EVs present in FBS, EV depletion from FBS using ultracentrifugation and starvation procedures are mainly used to eliminate FBS during MSC-EV collection [46]. However, the use of these methods does not exclude the presence of proteins and other animal-derived impurities present in FBS and does not take into account the limited EV production yield under starvation conditions. To overcome these limitations, we used MSC cells and chemically defined, substance-free media derived from nonhuman species for EV isolation [47,48]. 

MSC-EVs exert their antifibrotic effects through several potential mechanisms. MSC-EVs can deliver a variety of bioactive molecules, including proteins, lipids, and RNAs, which have been shown to influence cellular reprogramming and promote hepatocyte differentiation, thereby enhancing liver regeneration [49]. Another cellular target of MSC-EVs in the liver are HSCs, which play a pivotal role in liver fibrogenesis [3]. The activation of HSCs stands out as a key event in the progression of liver fibrosis [4]. Previous studies have demonstrated that EVs isolated from amnion-derived MSCs [50] and from placenta-derived MSCs [22] inhibit the activation of HSCs both in vivo and in 2D and 3D in vitro models of liver fibrosis. We previously demonstrated that EVs derived from HLSCs and BM-MSCs attenuated the activated phenotype of HSCs in vitro [21]. Consistent with these findings, our study revealed that treatment with EVs derived from both BM-MSCs and UC-MSCs cultured in a chemically defined medium resulted in the downregulation of fibrotic marker expression in activated HSCs.

The recent advancement of 3D culture systems has enabled the study of complex interactions between various cell types involved in fibrosis progression, especially hepatocytes and HSCs. Human primary hepatocytes are regarded as the gold standard for hepatology research [51]. However, their application is restricted by the limited availability of liver tissue, high costs, short lifespan in culture, and significant variability between different preparations [52]. In contrast, cell lines provide advantages such as availability, robustness, and reproducibility. HepG2 cells, for instance, are widely used in hepatotoxicity studies, but they are unable to detect metabolism-mediated hepatotoxicity due to the absence of P450 cytochrome enzymes [53]. Recently, Upcyte technology allowed the creation of human hepatocytes capable of proliferation while retaining certain differentiated functions [54].

In our study, we established two different liver 3D spheroids, incorporating hepatocyte surrogate cell line (HepG2) and HSCs, or UpHep. In both 3D models, we observed that the liver spheroids self-assembled 24 h post-seeding and maintained structural integrity up to 6 days post-seeding. Treatment with TGF-β1 resulted in the upregulation of fibrotic markers in spheroids formed by HepG2 and LX-2 cells and in spheroids generated by UpHep, indicative of a transition from a normal to a fibrotic phenotype.

The morphology of UpHep demonstrated significant heterogeneity, indicating a mixed population that includes not only mature hepatocytes but also other liver cells with a fibroblast-like shape. Moreover, the increased expression of profibrotic genes in UpHep spheroids upon TGF-β1 stimulation suggests that UpHep is not a pure culture of primary hepatocytes and may contain HSCs. Consequently, adding LX-2 cells to UpHep spheroids is unnecessary, as they may be inherently present in the culture. In contrast, the TGF-β1 stimulation of spheroids formed by HepG2 does not result in a profibrotic phenotype, making the addition of LX-2 cells essential. 

Then, we tested the impact of EVs derived from BM-MSCs and UC-MSCs on liver spheroids containing HepG2 and LX-2 cells, as well as those formed by UpHep. Our observations revealed that the EVs isolated from both types of MSCs suppressed the expression of fibrotic markers induced by TGF-β1. These findings suggest that MSC-derived EVs hold significant potential for mitigating fibrosis, aligning with a previous study that reported the capacity of EVs obtained by placental MSCs to suppress the TGF-β1-induced expression of fibrotic markers in liver organoids [22]. The 3D experimental settings corroborate the in vivo studies that showed the therapeutic use of MSC-EVs as a promising treatment for chronic liver disease [55]. 

## 5. Conclusions

In summary, the administration of EVs derived from BM-MSCs and UC-MSCs demonstrated antifibrotic effects in liver spheroids. These therapeutic effects are likely attributed to the inactivation of HSCs, as evidenced by reduced collagen production and αSMA expression. Therefore, this cell-free therapy presents itself as a promising alternative for the treatment of chronic liver disease. Additionally, the TGF-β1-induced liver fibrotic spheroid model holds potential as a valuable tool for antifibrosis drug screening.

## Figures and Tables

**Table 1:** Primers used in RT-PCR.

Gene	Forward Primer Sequence	Reverse Primer Sequence
*ACTA2*	TGGCTATTCCTTCGTTACTACTGCT	CTCATTTTCAAAGTCCAGAGCTACAT
*ALB*	TTATGCCCCGGAACTCCTTT	ACAGGCAGGCAGCTTTATCAG
*COL1A1*	CAAGAGGAAGGCCAAGTCGAG	TTGTCGCAGACGCAGATCC
*CYP1A1*	GGGCGTTCTGTCTTTGTAA	TGGGTTGACCCATAGCTTCT
*CYP3A4*	AATCTGTGCCTGAGAACACCAGA	AGTCCATTGGATGAAGCCCA
*TBP*	TGTGCACAGGAGCCAAGAGT	ATTTTCTTGCTGCCAGTCTGG
*TGFB1*	GACTACTACGCCAAGGAGGT	GGAGCTCTGATGTGTTGAAG

**Table 2:** Antibodies used for protein detection.

Antibody	Code	Source
Primary anti-Alix	CST#2171	mouse
Primary anti-CD9	CST#13174	rabbit
Primary anti-CD63	CST#52090	rabbit
Primary anti-CD81	CST#56039	rabbit
Primary anti-GM130	CST#12480	rabbit
Primary anti-TSG101	CST#72312	rabbit
Secondary anti-mouse	Invitrogen #31430	goat
Secondary anti-rabbit	Invitrogen #31462	goat

## Data Availability

The original contributions presented in this study are included in the article/Supplementary Materials; further inquiries can be directed to the corresponding author/s.

## Supplementary Materials

The following supporting information can be downloaded at https://www.mdpi.com/article/10.3390/biomedicines12081849/s1: Figure S1: Expression of Collagen I in liver spheroids; Figure S2: characterization of UpHep; Figure S3: effect of EVs on the morphology and functionality of liver spheroids treated with TGF-β1.
