# Supplementary material for: Mesenchymal Stromal Cell-Derived Extracellular Vesicles for Reversing Hepatic Fibrosis in 3D Liver Spheroids
Source: Biomedicines. 2024 Aug 14;12(8):1849. doi: 10.3390/biomedicines12081849 (PMC11351945; doi:10.3390/biomedicines12081849)
Supplement: Supplementary file 1 [file biomedicines-12-01849-s001.zip › biomedicines-3111960-supplementary.pdf]

biomedicines

MDPI

Article

Mesenchymal Stromal Cell-Derived Extracellular Vesicles for Reversing Hepatic Fibrosis in 3D Liver Spheroids

Giulia Chiabotto, Armina Semnani, Elena Ceccotti, Marco Guenza, Giovanni Camussi and Stefania Bruno *

Supplementary materials:

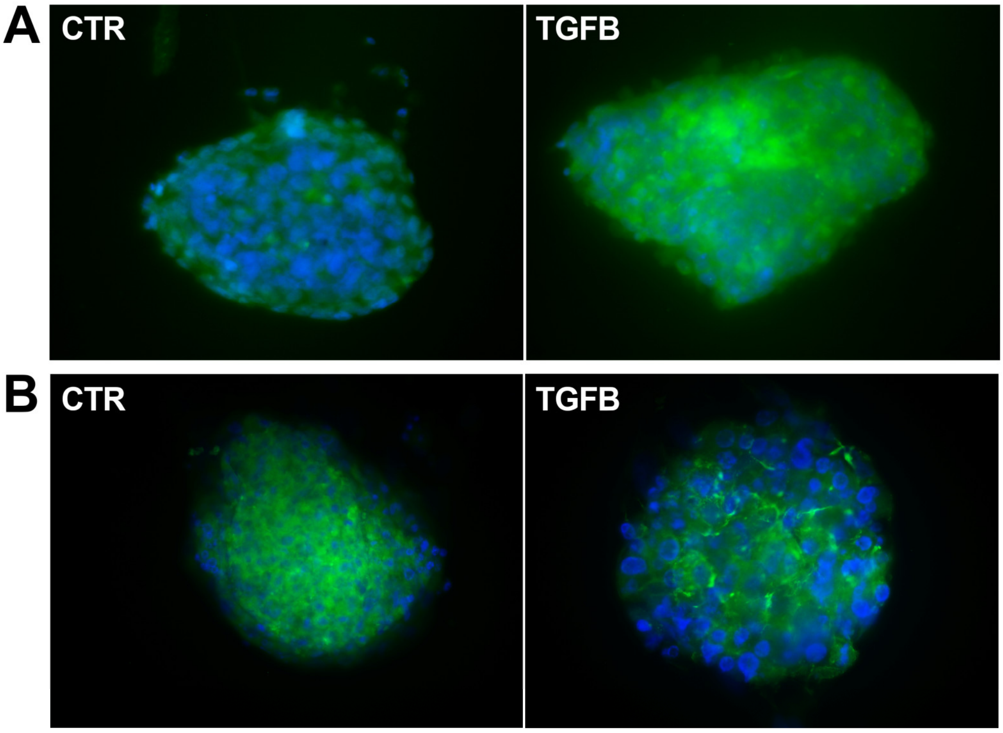

Figure S1. Expression of Collagen I in liver spheroids. (**A,B**) Fluorescence microscopy analysis of liver organoids formed by the co-culture of HepG2 and LX-2 (**A**) and by UpHep alone (**B**) on day 6 after cell seeding. On the left are control spheroids, cultured in the absence of TGF-β1, while on the right are spheroids activated following stimulation with TGF-β1 (original magnification: 20×).

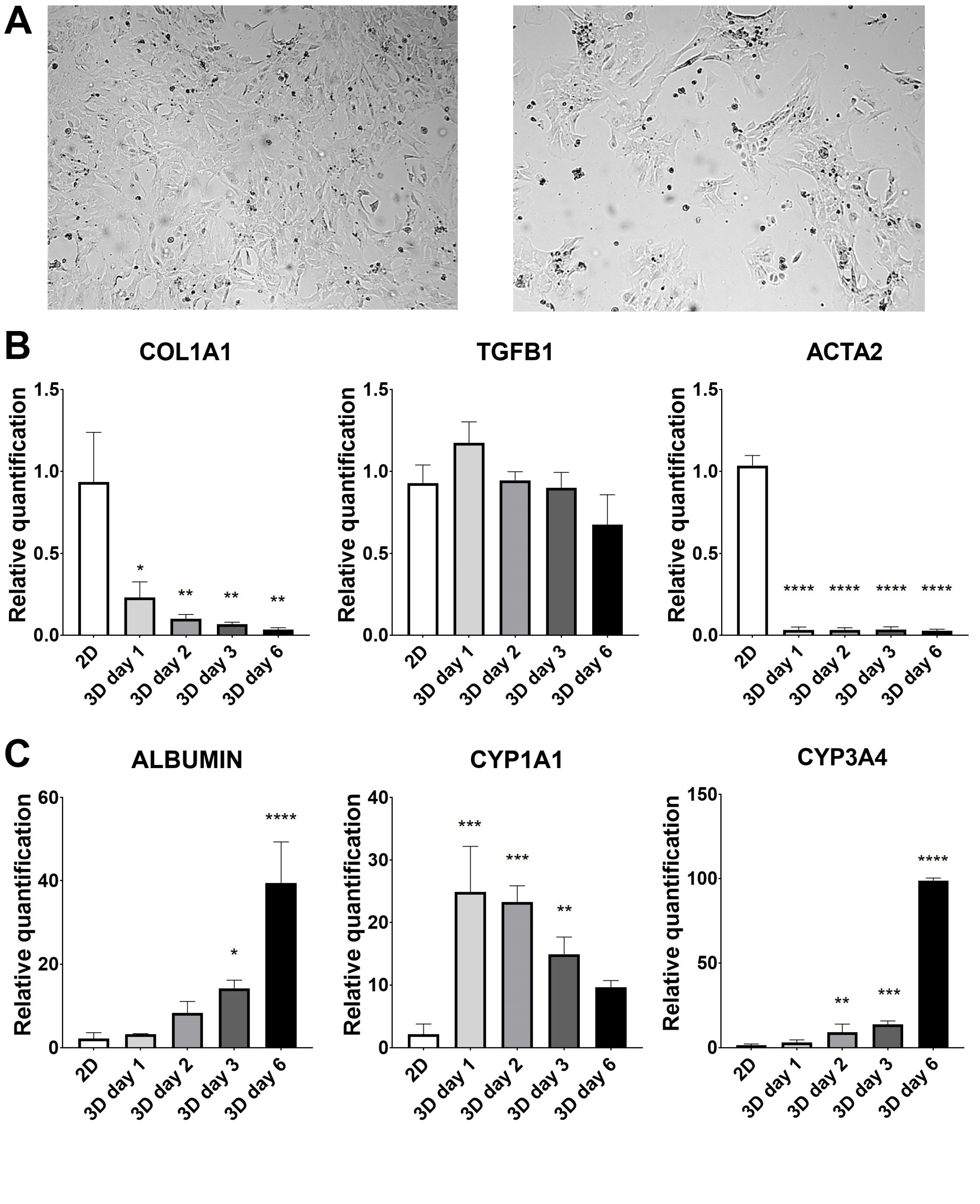

Figure S2. Characterization of UpHep. (**A**) Morphological observation under a light microscope of UpHep in 2D culture at passage 0 (left) and passage 1 (right). Original magnification: 10×. (**B**,**C**) Quantitative real-time PCR analysis of the expression of pro-fibrotic genes (**B**) and hepatocyte-specific genes (**C**) in UpHep cultured in 2D or in 3D up to 6 days after seeding. Gene expression levels were normalized to those of the *TBP* control gene. UpHep cultured in 2D were used as reference control. Statistical analysis was conducted on data obtained from at least three independent experiments using a two-way ANOVA test: * $p < 0.05$; ** $p < 0.01$; *** $p < 0.001$; **** $p < 0.0001$.

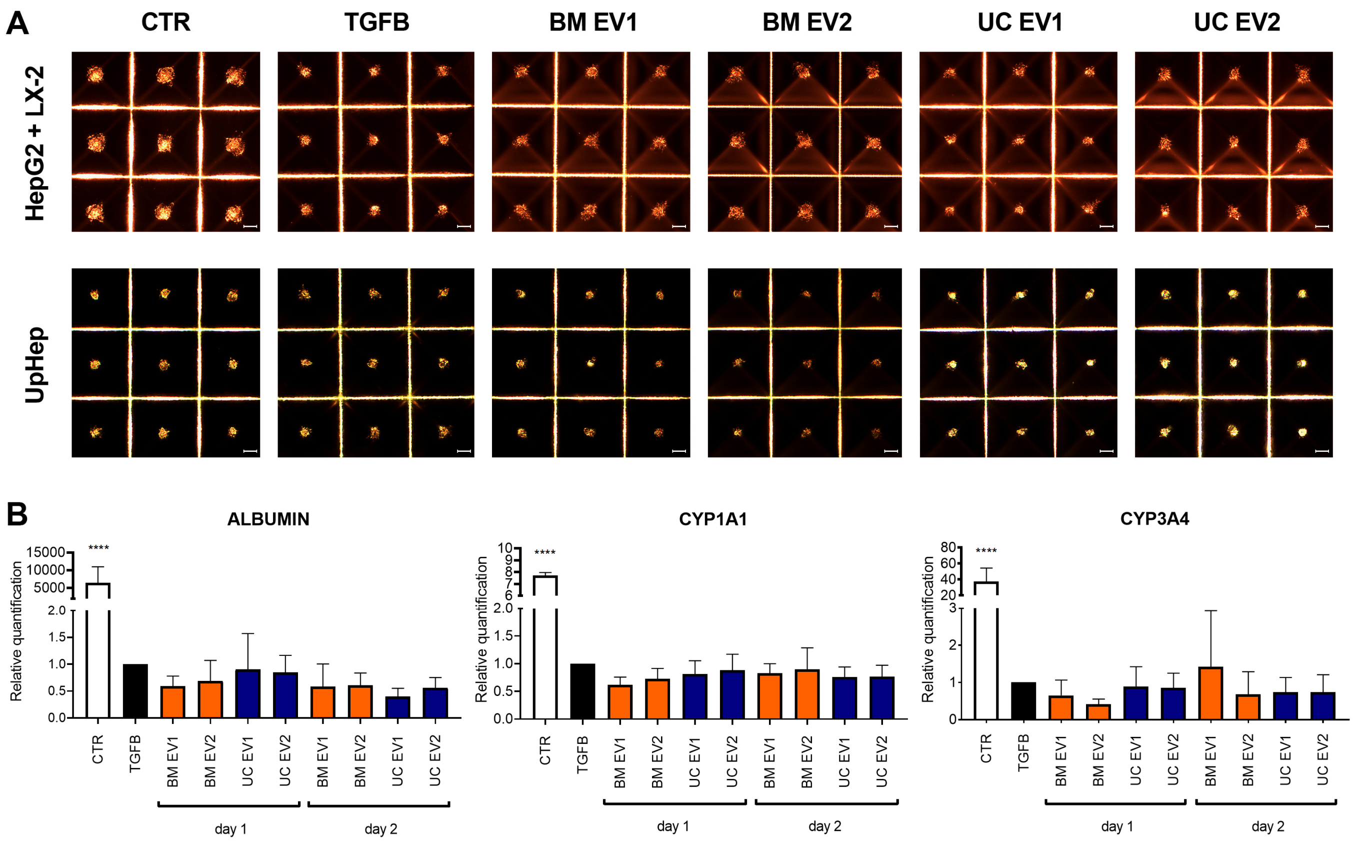

Figure S3. Effect of EVs on the morphology and functionality of liver spheroids treated with TGF-β1. (**A**) Morphological observation under a light microscope of liver organoids generated from HepG2 and LX-2 cells, or from UpHep, on day 6 after seeding (scale bar, 100 μm). In the figures shown, EVs isolated from BM-MSCs and UC-MSCs were administered 24 hours after stimulation with TGF-β1 (EV1 = 2×10^8 EVs/well and EV2 = 1×10^9 EVs/well). (**B**) Quantitative real-time PCR analysis of the expression of hepatocyte-specific genes in liver spheroids formed by UpHep 6 days after seeding. Gene expression levels were normalized to those of the TBP control gene. UpHep spheroids activated by TGF-β1 were used as a reference control, while spheroids not activated by TGF-β1 were used as a negative control. Statistical analysis was conducted on data obtained from at least three independent experiments using a two-way ANOVA test: **** $p < 0.0001$.

Disclaimer/Publisher's Note: The statements, opinions and data contained in all publications are solely those of the individual author(s) and contributor(s) and not of MDPI and/or the editor(s). MDPI and/or the editor(s) disclaim responsibility for any injury to people or property resulting from any ideas, methods, instructions or products referred to in the content.
